# Magnetic resonance-guided radiation therapy: the beginning of a new era

**DOI:** 10.1186/s13014-020-01599-z

**Published:** 2020-07-08

**Authors:** Guillaume Landry, Stefanie Corradini, Claus Belka

**Affiliations:** Department of Radiation Oncology, University Hospital, LMU Munich, Munich, Germany

The widespread use of volumetric image guidance in external beam radiation therapy can be traced back to the development of x-ray flat panel technology, which allowed the introduction of cone beam computed tomography in the early 2000s. The ability to verify patient positioning in 3D has come hand in hand with advances in dose conformity from the development of techniques such as IMRT and VMAT. However, poor CBCT contrast frequently limits visualization of the target to surrogate features, such as the bony anatomy or implanted fiducial markers, and does not readily permit soft tissue and lesion visualization in all cases. This implicitly limits our ability to perform fine-grained treatment plan adaptation on a fraction basis with more advanced modifications than simple couch shifts.

While the superior soft tissue contrast of magnetic resonance imaging solves the lesion visualization issue, its use for in-room imaging and plan adaptation is far from trivial. One needs to account for the interplay of the magnetic field and the linear accelerator, as well as for the dose distortions associated with the Lorentz force on secondary electrons. The advancements in MR guided radiotherapy devices, and the recent installation of several MR-linac systems throughout the world, are thus highly impressive given the challenges they tackled. Online treatment plan adaptation, relying on electron density estimation from MRI images, on the fly segmentation, treatment optimization and quality assurance is now performed clinically at several institutions. The high complexity of performing online adaptation safely requires a multi-disciplinary team of radiation oncologists, technologists and physicists to perform these tasks.

In this special issue of Radiation Oncology dedicated to the latest advancements in MR guided radiotherapy, a collection of review and research articles highlights the status of the field and discusses recent challenges, but also presents potential future directions. In Corradini et al. [1], clinical experts lay out the current clinical applicability for early adopters of MR guided radiotherapy systems, and the potential new perspectives from the technology and benefit to patients. Kurz and colleagues [2] present the technical state of the art of current systems and of MR guided adaptive radiotherapy, as well as the challenges medical physicists and engineers aim at tackling in the near future. With some delay, proton therapy has also adopted CBCT image guidance at latest generation treatment rooms. Hoffmann et al. [3] discuss how a similar adoption of MR guidance may be realized, accounting for the stringent requirements on proton range calculation, and the obvious challenge of accounting for proton deflection by the Lorentz force in treatment planning, as well as the technical challenges for accelerator physicists.

We are still in the early days of MR-guided radiation therapy, a technology with an impressive perceived potential. Online treatment plan adaptation is forcing us to reconsider paradigms of radiation therapy and to re-imagine clinical workflows. Of note, the concurrent explosion of deep learning success stories in medical imaging may strongly impact the field of MR guided radiotherapy. We hope that this special issue of Radiation Oncology sheds some light on potential paths forward and the current state of the field in general, as well as focussing minds on avoiding the pitfalls attendant to such new technology.

## References

[1] Corradini S, Alongi F, Andratschke N, Belka C, Boldrini L, Cellini F (2019). MR-guidance in clinical reality: current treatment challenges and future perspectives. Radiat Oncol.

[2] Kurz C, Buizza G, Landry G, Kamp F, Rabe M, Paganelli C (2020). Medical physics challenges in clinical MR-guided radiotherapy. Radiat Oncol.

[3] Hoffmann A, Oborn B, Moteabbed M, Yan S, Bortfeld T, Knopf A (2020). MR-guided proton therapy: a review and a preview. Radiat Oncol.

